# Immune Response to SARS-CoV-2 XBB.1.5 and JN.1 Variants Following XBB.1.5 Booster Vaccination in Liver Transplant Recipients

**DOI:** 10.3390/v16121942

**Published:** 2024-12-19

**Authors:** Philippa von der Schulenburg, Georg M. N. Behrens, Markus Hoffmann, Alexandra Linke, Inga Nehlmeier, Amy Madeleine Kempf, Metodi Stankov, Marc Lütgehetmann, Jacqueline Jahnke-Triankowski, Marylyn M. Addo, Lutz Fischer, Ansgar W. Lohse, Stefan Pöhlmann, Julian Schulze zur Wiesch, Martina Sterneck

**Affiliations:** 1 I. Department of Internal Medicine, University Medical Center Hamburg-Eppendorf, 20246 Hamburg, Germany; p.vonderschulenbur@gmail.com (P.v.d.S.); a.linke@uke.de (A.L.); m.addo@uke.de (M.M.A.); alohse@uke.de (A.W.L.); sterneck@uke.de (M.S.); 2 Department of Rheumatology and Immunology, Hannover Medical School, 30625 Hannover, Germany; behrens.georg@mh-hannover.de (G.M.N.B.); stankov.metodi@mh-hannover.de (M.S.); 3 German Center for Infection Research (DZIF), Partner Site Hannover-Braunschweig, 38124 Braunschweig, Germany; 4 Infection Biology Unit, German Primate Centre-Leibniz Institute for Primate Research, 37077 Göttingen, Germany; mhoffmann@dpz.eu (M.H.); inehlmeier@dpz.eu (I.N.); akempf@dpz.eu (A.M.K.); spoehlmann@dpz.eu (S.P.); 5 Faculty of Biology and Psychology, Georg-August-University Göttingen, 37073 Göttingen, Germany; 6 German Center for Infection Research (DZIF), Partner Site Hamburg-Lübeck-Borstel-Riems, 38124 Braunschweig, Germany; mluetgehetmann@uke.de; 7 Institute of Medical Microbiology, Virology and Hygiene, University Medical Center Hamburg-Eppendorf, 20246 Hamburg, Germany; 8 Department of Visceral Transplantation, University Medical Center Hamburg-Eppendorf, 20246 Hamburg, Germany; j.jahnke-triankowski@uke.de (J.J.-T.); lfischer@uke.de (L.F.); 9 University Transplant Center, University Medical Center Hamburg-Eppendorf, 20246 Hamburg, Germany; 10 Institute for Infection Research and Vaccine Development (IIRVD), University Medical Center Hamburg-Eppendorf, 20246 Hamburg, Germany

**Keywords:** SARS-CoV-2, vaccination, COVID-19, liver transplantation, XBB.1.5, JN.1, immunosuppression, cellular immune response, humoral immune response

## Abstract

Background/Objectives: The efficacy of monovalent BNT162b2 Omicron XBB.1.5 booster vaccination in liver transplant recipients (LTRs) has yet to be described, particularly regarding the immune response to emerging variants like JN.1. Methods: This study evaluated humoral and cellular immune responses in 34 liver transplant recipients (LTRs) with varying SARS-CoV-2 immune histories before and after receiving a BNT162b2 Omicron XBB.1.5 booster vaccination. The assessment involved variant-specific serology, pseudovirus neutralization tests, and Interferon-γ release assays. Results: Participants had a median of four prior vaccinations, with 91.2% having a history of infection. Post-vaccination, significant increases in both Wuhan anti-S and Omicron-specific IgG antibodies and improved neutralization of B.1, XBB.1.5, and JN.1 pseudovirus particles were observed. Also, T-cell responses significantly increased post-vaccination. However, 17.6% of LTRs had no neutralizing antibodies against XBB.1.5 and JN.1, while 100% of healthy controls did. Shortly after vaccination, 18% of patients developed mild COVID-19. These LTRs had particularly low immune responses at baseline. Conclusions: The monovalent XBB.1.5 booster improved overall SARS-CoV-2-specific immunity. However, some LTRs still showed low or undetectable immune responses, indicating that ongoing monitoring and further booster doses are necessary in this high-risk group.

## 1. Introduction

Vaccination has played a crucial role in reducing severe COVID-19 cases, hospitalizations, and deaths worldwide [1]. However, liver transplant recipients (LTRs) are still a vulnerable population and exhibit a significantly impaired immune response to SARS-CoV-2 vaccination [2,3,4], achieving only partial immunity even after multiple booster doses [5,6,7,8,9,10]. With waning immunity and the emergence of new immune escape variants, the protection level from previous SARS-CoV-2 vaccinations and infections remains uncertain but crucial for this high-risk populations. Data indicate that monovalent XBB.1.5-adapted vaccines elicit a strong immune response in healthy individuals, effectively targeting XBB.1.5 [11]. On the other hand, the immune protection achieved by previous infections and variant-adapted vaccinations against the currently dominant variants, such as JN.1, KP.2, KP.3, and XEC, remain uncertain [11,12,13]. JN.1, a BA.2.86 subvariant, has rapidly become predominant and is highly immune-evasive [12,13]. KP.2 and KP.3 have descended from JN.1 and differ only by a few mutations in spike protein. XEC, a recombinant variant of KS.1.1 and KP.3.3, shows an enhanced humoral evasion and is foreseen to become a dominant subvariant [14,15]. For this winter, the European Medicines Agency (EMA) has recommended new vaccines targeting JN.1 [16,17]**.** Despite the importance of vaccination for high-risk LTRs, data on the impact of the XBB.1.5 vaccine on the immune response to the JN.1 variant are lacking. This small, prospective single-center study aimed to evaluate the immune response following a monovalent BNT162b2 XBB.1.5 vaccination in LTRs last winter, offering insights to improve future vaccination strategies for this vulnerable population.

## 2. Materials and Methods

We prospectively analyzed 34 adult LTRs who received a 30 µg dose of BNT162b2 Omicron XBB.1.5 (Raxtozinameran, BioNtech, Mainz, Germany) booster vaccination at the University Medical Center Hamburg-Eppendorf between September 2023 and January 2024. The vaccination followed the recommendations of the Robert Koch Institute (RKI), and COVID-19 vaccines were supplied by the Ministry of Health in Germany at the time of the study. The patients were consecutively enrolled during routine visits to our outpatient liver transplant unit based on their consent to participate in the study, which involved the collection of additional blood samples. Data were collected from questionnaires, electronic medical records and blood samples were taken before and four to six weeks post-vaccination.

This study was approved by the local ethics committee of Hamburg, Germany (Reg. numbers PV7103 and PV7298).

### 2.1. Assessment of the Humoral and Cellular Spike-Specific Immune Response

SARS-CoV-2 IgG was measured using quantitative Enzyme-linked Immunosorbent Assays (ELISAs) against the Wuhan Hu-1 (EI 2606-9601-10 G, EUROIMMUN, Lübeck, Germany), anti-Omicron (EI 2606-9601-30 G, EUROIMMUN, Lübeck, Germany), and Nucleocapsid protein (EI 2606-9601-2 G, EUROIMMUN, Lübeck, Germany), as previously described [11,18].

T-cell response was measured by a Wuhan-Hu-1 Spike-specific interferon-Gamma Release Assay (IGRA, EUROIMMUN, Lübeck, Germany) [6,7,9,18].

### 2.2. Production of Pseudovirus Particles (Pps) and Pseudovirus Neutralization Tests (pVNTs)

pVNTs were performed at the Infection Biology Unit of the German Primate Centre in Göttingen according to a previously published protocol with slight modifications [11,19]. Briefly, neutralization efficiency was assessed based on the relative pseudovirus entry inhibition, with signals generated using pseudovirus particles incubated in the absence of plasma serving as reference (=0% inhibition). A non-linear regression model was applied to calculate the neutralizing titer 50 (NT50), representing the plasma dilution necessary for achieving half-maximal inhibition.

### 2.3. Statistical Analysis

Descriptive statistics were presented as median (range) for continuous variables or number (percentage) for categorical variables. Neutralization titers were transformed into geometric mean titers (GMTs). SPSS statistics version 27 (IBM Corp, Armonk, NY, USA) was used for statistical analysis, employing Pearson’s chi-squared test, Fisher’s exact test, Mann–Whitney U Test, Wilcoxon signed-rank test, Kruskal–Wallis test, or Spearman’s rank correlation. Prism GraphPad 8.0.1 (Windows, Graph-Pad Software, San Diego, CA, USA) was used to create figures.

## 3. Results

### 3.1. Study Cohort and Patient Characteristics

Clinical and demographic details of the 34 LTRs included in this study are shown in Table 1. Most LTRs (median age: 56 years) were long-term recipients with a median of 9.5 (range: 0–28) years since transplantation. This real-world study cohort had a heterogeneous vaccination and infection status: the median number of prior vaccine doses was four (range: 2–5), and 91.2% (31/34) of patients had a previous SARS-CoV-2 infection; only 8.8% (3/34) had no history or serological evidence of a prior infection. Most patients had exclusively received mRNA vaccines, while only a few (*n* = 5) had additionally received a viral vector vaccine. Notably, all patients had received at least two doses of mRNA vaccine in the past. Blood samples were analyzed before and within a median of 33.5 days (range: 22–56) after vaccination. Of note, six patients developed confirmed COVID-19 with mild symptoms shortly after vaccination (median of 16 days after vaccination, range: 1–34) (Supplementary Table S1).

### 3.2. Humoral Immune Response Determined by ELISAs

**Figure 1:**
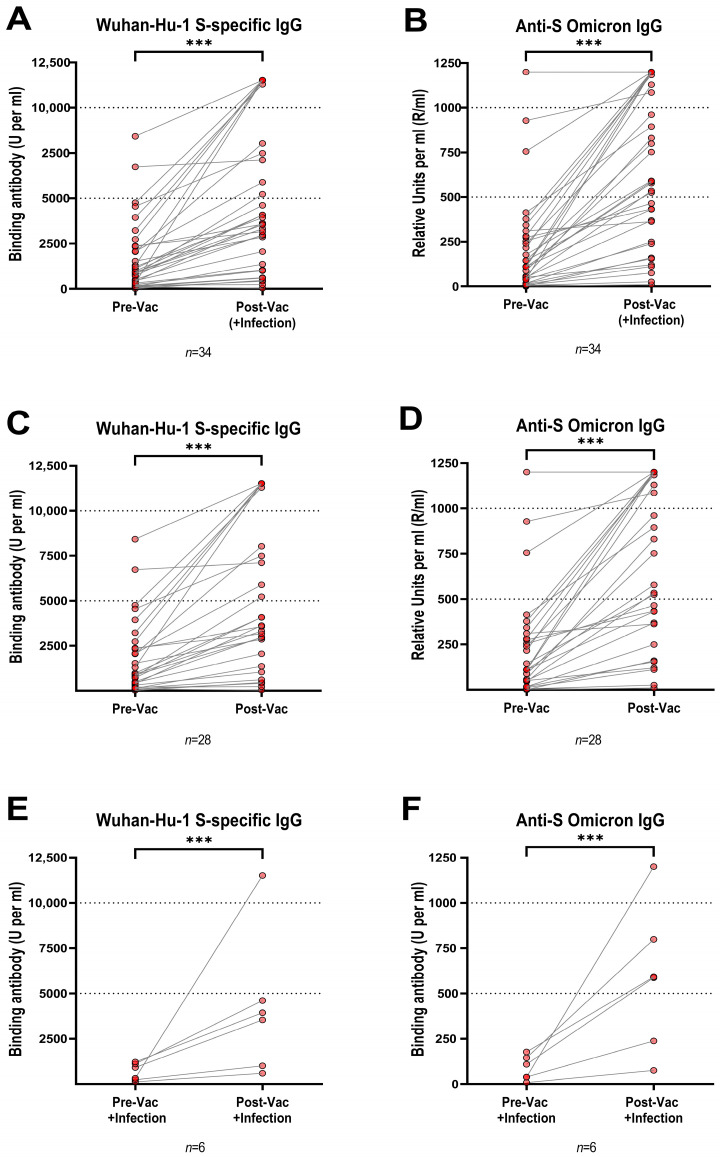
(**A**,**B**) Concentrations of Wuhan-Hu-1 S-specific IgG and Omicron S-specific IgG in plasma of all LTRs (*n* = 34) taken before and after vaccination with BNT162b2 Omicron XBB.1.5 vaccine or after vaccination and infection. (**C**,**D**) Concentrations of Wuhan-Hu-1 S-specific IgG and Omicron S-specific IgG in plasma in all LTRs with sole vaccination taken before and after vaccination with BNT162b2 Omicron XBB.1.5 vaccine (*n* = 28). (**E**,**F**) Concentrations of Wuhan-Hu-1 S-specific IgG and Omicron S-specific IgG in plasma in LTRs with breakthrough infection shortly after vaccination taken before vaccination with BNT162b2 Omicron XBB.1.5 vaccine and after vaccination and infection (*n* = 6). Paired T-test performed statistical significance (*** = *p* < 0.001). Red dots represent individual LTRs, grey lines connect paired data points for the same LTR. Abbreviations: IgG: immunoglobulin G; Vac: vaccination; S: spike.

We determined the Wuhan-Hu-1 anti-S and the anti-Omicron IgG titers before and after XBB.1.5 vaccination (Figure 1) in all LTRs (*n* = 34, Figure 1A,B), in LTRs who did not have a breakthrough infection shortly after vaccination (*n* = 28, Figure 1C,D), and in LTRs with breakthrough infection shortly after vaccination (*n* = 6, Figure 1E,F). A strong correlation (Spearman r = 0.98, *p* < 0.001, Supplementary Figure S1A) was found between both assays. At baseline, 1/34 (2.9%) and 3/34 LTRs (8.8%) were negative for Wuhan anti-S and anti-Omicron IgG, respectively. At baseline, the median Wuhan anti-S and anti-Omicron IgG levels of all LTRs were 909.5 BAU/mL and 109.3 RU/mL (Figure 1A,B), respectively, which is slightly lower compared to our previously published cohort of 65 healthcare workers (HCWs) (mean: Wuhan anti-S IgG: 1422 BAU/mL, anti-Omicron IgG: 199 RU/mL) [11]. Post-vaccination, all 28 LTRs without additional infection tested positive for Wuhan anti-S IgG and all but one for anti-Omicron IgG (Figure 1C,D). Also, a significant 3.8-fold change in Wuhan anti-S IgG titers (945 to 3595 BAU/mL; *p* < 0.0001) and a 5.1-fold change in anti-Omicron IgG levels (from 110 to 555.6 RU/mL; *p* < 0.0001) were seen in this group. In comparison, there was a slightly less pronounced increase in our previously published cohort of HCWs (2.9 and 3.6-fold change for Wuhan and Omicron anti-S IgG, respectively) [11].

For the six LTRs with infections shortly after vaccination (Figure 1E,F), baseline titers were very low (613 BAU/mL and 74 RU/mL for Wuhan and Omicron anti-S, respectively). Still, after vaccination and infection, they showed a strong 6.1-fold and 8.0-fold increase in Wuhan and Omicron anti-S levels. This resulted in similar median titers (3735 vs. 3595 BAU/mL and 581 vs. 555.5 RU/mL for Wuhan anti-S and anti-Omicron IgG, respectively) compared to those in booster-vaccinated LTRs without breakthrough infection.

Notably, the three LTRs without any prior infection had significantly lower median IgG levels both before (131 vs. 978 BAU/mL; *p* = 0.014 and five vs. 111 RU/mL; *p* = 0.005 for Wuhan anti-S and anti-Omicron IgG, respectively) and after vaccination (226 vs. 3934 BAU/mL; *p* = 0.007 and 26 vs. 591 RU/mL; *p* = 0.009 for Wuhan anti-S and anti-Omicron IgG, respectively) compared to previously infected LTRs (Supplementary Table S2, Supplementary Figure S2).

### 3.3. Neutralization of Pseudovirus Particles

**Figure 2:**
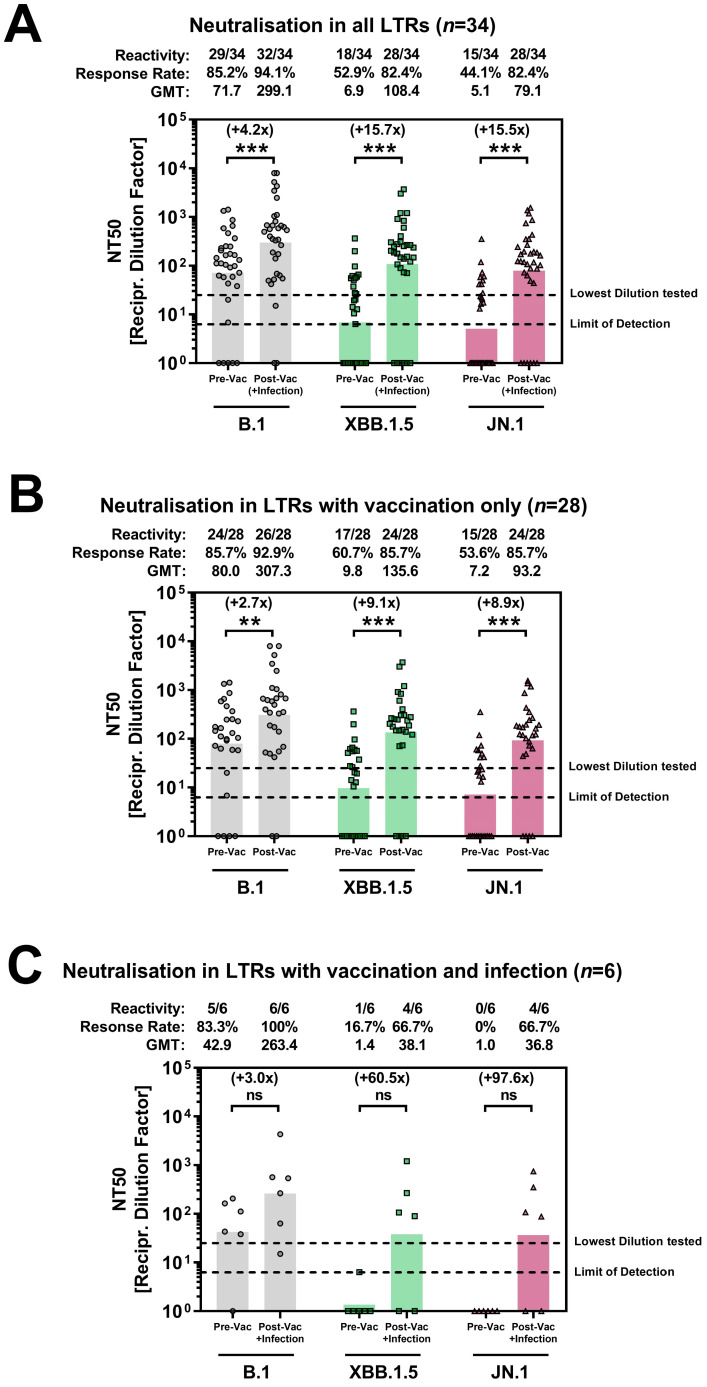
(**A**–**C**) Analysis of neutralization capacity of antibodies before and after XBB.1.5 vaccination or vaccination and infection against B.1, XBB.1.5, and JN.1 in LTRs. Wilcoxon signed-rank test assessed statistical significance (ns = *p > 0.05;* ** = *p* < 0.01; *** = *p* < 0.001). For graphical reasons, plasma samples below the limit of detection were set at bottom of axis. Grey dots, green squares, and purple triangles represent the individual responses of LTRs to B.1, XBB.1.5, and JN.1, respectively. Columns indicate the geometric mean NT50 values. Abbreviations: LTRs: liver transplant recipients; GMT: geometric mean titer; Recipr: reciprocal.

Neutralization was analyzed using pseudovirus particles (pps) with SARS-CoV-2 spike proteins from the B.1, XBB.1.5, and JN.1 lineages. Baseline response rates (*n* = 34) were 85% for B.1 pps but only 53% and 44% for XBB.1.5 pps and JN.1 pps, respectively (Figure 2A). Also, XBB.1.5 pps and JN.1 pps were less efficiently neutralized than B.1 pps (GMTs for B1, XBB.1.5 and JN.1 of 71.7, 6.9, and 5.1, respectively). In comparison, 100% of HCWs responded to B.1 pps and 55% to XBB.1.5 pps with higher GMTs (1597 and 28, respectively) [11]. After XBB.1.5 vaccination, response rates in LTRs without additional vaccination (*n* = 28) increased to 93%, 86%, and 86% for B.1 pps, XBB.1.5 pps, and JN.1 pps, respectively (Figure 2B). For B.1 pps, there was a 2.7-fold increase in GMTs (from 80 to 307.3, *p* < 0.001), while increases were higher for XBB.1.5 pps (9.1-fold, from 9.8 to 135.6, *p* < 0.001) and JN.1 pps (8.9-fold, from 7.2 to 93.2, *p* < 0.001). Notably, none of the three LTRs without prior SARS-CoV-2 infection responded to XBB.1.5 pps or JN.1 pps, and only one-third responded weakly to B.1 pps after vaccination (Supplementary Figure S3). Altogether, there was a strong correlation between the neutralization of XBB.1.5 pps and JN.1 pps (Spearman r = 0.973, *p* < 0.001, Supplementary Figure S1B).

Among the six LTRs with COVID-19 after vaccination (Figure 2C), baseline neutralization was 83% for B.1 pps but significantly lower, with only 16.7%, for XBB.1.5 pps and 0% for JN.1 pps compared to LTRs without infection after vaccination. Post-vaccination and infection, all six LTRs (100%) neutralized B.1 pps with a 3.0-fold change in GMTs (42.9 to 263.4). Neutralization capacities for XBB.1.5 pps and JN.1 pps increased, with a response rate of 67% for both pseudo particles and GMT fold changes of 60.5 and 97.6, respectively.

Altogether, post-vaccination 17.6% (6/34) of all LTRs showed no response to XBB.1.5 pps and JN.1 pps, while all HCWs did [11]. Notably, non-responding LTRs had markedly lower antibody levels compared to responding LTRs, with highly significant differences observed for both Wuhan anti-S and anti-Omicron IgG (128.2 BAU/mL vs. 1161 BAU/mL, *p* = 0.001, and 6.7 AU/mL vs. 144 AU/mL, *p* = 0.001, respectively). Similarly, the lower rate of previous infections was also highly significant in non-responding LTRs (50% vs. 100%, *p* = 0.003; Supplementary Table S3).

### 3.4. Spike-Specific T-Cell Response Measured by IGRA

**Figure 3:**
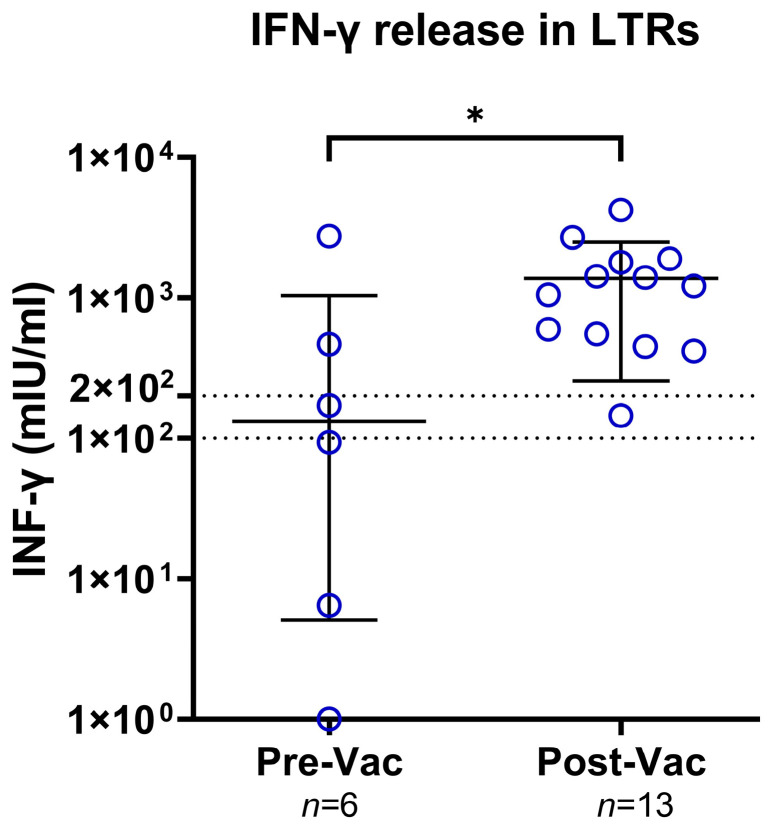
Scattergram with individual interferon-gamma (IFN- γ) levels before and after XBB.1.5 vaccination. The Mann–Whitney Test performed statistical analysis (* = *p* < 0.05). The blue circles represent IFN- γ levels in individual LTRs. Solid lines represent median and interquartile range. Dotted lines represent cut-off values with interferon-gamma (IFN-γ) levels of <100 mlU/mL being negative, 100–200 mlU/mL being low positive, and >200 mlU/mL being high-positive. Abbreviations: LTRs: liver transplant recipients; IFN- γ: interferon-gamma; Vac: vaccination.

Altogether, six LTRs were studied before and thirteen after XBB.1.5 booster vaccination (Figure 3). Before vaccination, two patients showed no T-cell response after spike-specific stimulation, while afterward, all LTRs showed a positive IFN-γ release. The median IFN-γ levels rose from 132.3 mlU/mL to 1210 mlU/mL (*p* = 0.046). One LTR without prior infection was assessed and showed a negative result before and a low positive result (<200 mlU/mL) after vaccination.

## 4. Discussion

Here, we investigated LTRs’ humoral and cellular immune responses following XBB.1.5 booster vaccination in the fall and early winter of 2023 to evaluate their potential protection against different SARS-CoV-2 variants, particularly the JN.1 variant.

Overall, in this real-world cohort of LTRs with a median of four prior vaccinations and a prior infection rate of 91.2%, the XBB.1.5 booster vaccination led to a strong increase in humoral and cellular immune response. In vitro neutralization rates showed increased response rates against all variant particles, including B.1, XBB.1.5, and JN.1. However, 17.6% of LTRs (6/34) still showed no neutralizing antibody response against XBB.1.5 pps and JN.1 pps after vaccination.

We used two ELISAs to determine spike-specific IgG antibodies against Wuhan and Omicron variants. Both assays showed a strong correlation and high favorable rates of detectable antibodies even at baseline in LTRs (Wuhan anti-S 97.1% and anti-Omicron IgG 91.2%), with median titers slightly lower than those in HCWs [11]. This may suggest a similar baseline humoral immune response with LTRs having had more previous vaccine doses (median five vs. four) and more prior infections (91.2% vs. 81.5%).

However, for this in-depth analysis, we also conducted the neutralization assay, which demonstrated weaker humoral protection rates, with 14.8% showing no response to B.1 pps at baseline, compared to 100% of HCWs showing a response [11]. Also, despite the high rate of prior SARS-CoV-2 infections, the baseline response rate to XBB.1.5 pps was only 52.9%, with a low GMT of 6.9, most likely due to a waning immune response [5,20]. The response rate to JN.1 pps was lower, with 44.1% and a GMT of 5.1. After vaccination, LTRs showed a reasonable response rate against both XBB.1.5 pps (82.4%) and JN.1 pps (82.4%), but with reduced neutralization capacities observed for JN.1 pps. Therefore, our findings and other studies [16,17,21,22] emphasize the need for vaccines targeting the JN.1 family, as recommended by regulatory agencies.

Six LTRs developed mild COVID-19 shortly post-vaccination, most likely from the Omicron variants EG.5 and BA.2.86 or its sublineage JN.1 [23,24]. At baseline, only one patient showed an immune response to XBB.1.5 pps and none to JN.1 pps. Also, the Wuhan anti-S and anti-Omicron antibody titers were all below a threshold of approximately 1200 BAU/mL and 180 AU/mL, respectively (Supplementary Table S1), underscoring their susceptibility to COVID-19. In two patients, this lacking response persisted even after breakthrough-infection.

A definitive threshold for antibody titers that confer protection against COVID-19, whether against infection or severe illness, has not yet been established. Longitudinal studies with clinical endpoints are needed to determine such a threshold. However, the continuous emergence of new variants adds ongoing uncertainty regarding the effectiveness of immune protection against current strains [7,11,15].

In LTRs, hybrid immunity, resulting from both previous infection and vaccination, has been shown to enhance immune responses [10,25]. Similarly, our findings underscore the importance of hybrid immunity, as the three previously uninfected LTRs exhibited particularly low antibody levels and showed no neutralization capacity against the XBB.1.5 and JN.1 variants, even after vaccination.

This is the first study to investigate LTRs’ immune response following a BNT162b2 Omicron XBB.1.5 booster vaccination. Our findings align with studies on other immunocompromised populations, which have demonstrated enhanced neutralizing responses to XBB.1.5 pps and JN.1 pps after booster vaccination [21,26]. However, Johnston et al. reported a decline in XBB.1.5 neutralization capacity within three months after receiving a bivalent booster in solid organ transplant recipients, which was restored by a second booster dose [27].

In 2023, vaccination recommendations in Germany specifically called for the use of a monovalent variant-specific vaccine rather than a bivalent one. The monovalent booster demonstrated strong efficacy, as evidenced by the increased humoral and cellular immune responses and improved neutralization capacity in LTRs. Here, we demonstrate that the XBB.1.5-specific booster not only enhanced the humoral immune response against the wild-type Wuhan strain in LTRs but is particularly effective in enhancing the immune response against the XBB.1.5 and also the newer JN.1 variant (fold changes of 2.7, 9.1, and 8.9 for B.1, XBB.1.5 pps, and JN.1 pps, respectively). In agreement with our data, other studies have shown favorable outcomes of monovalent boosters. For instance, research comparing monovalent and bivalent mRNA vaccines found that the monovalent XBB.1.5 booster elicited significantly higher neutralizing antibody titers against the JN.1 variant compared to bivalent boosters targeting earlier variants such as BA.1 or BA.4–5 [28]. Initial studies in immunocompetent individuals concerning the monovalent vaccines based on the spike protein of JN.1 also show enhanced immune responses to JN.1, the Wuhan strain, and newly emerging variants [29,30].

Limitations of this study include the small, single-center cohort design, which may limit the generalizability of our findings to broader populations. Also, T-cell responses were only determined against the Wuhan variant. Furthermore, as a real-world study, the patients had heterogeneous vaccination and infection statuses at baseline, which might limit the comparability of the immune response findings. However, as vaccination strategies evolve, evaluating immune responses in homogeneous cohorts becomes almost impossible due to numerous influencing factors, such as prior vaccine types, varying numbers of doses, differing intervals since the last antigen exposure, diverse infection histories in terms of the number and variants of infections, and individual risk factors or immunosuppressive regimens. Studying a real-life cohort offers valuable insights into the full spectrum of immune responses, providing a comprehensive understanding of the collective immune status in this vulnerable population.

## 5. Conclusions

In conclusion, our study revealed a lower baseline humoral immune protection in LTRs compared to HCW. The monovalent XBB.1.5 booster dose significantly enhanced immune responses with a stronger response to variants, including the newer JN.1 strain than to the wild type strain. However, approximately one in six LTRs still showed no response to XBB.1.5 and JN.1 variants after booster vaccination. Therefore, ongoing monitoring and variant-specific booster strategies are crucial to optimize future protection in this vulnerable group. Further studies with clinical endpoints are needed to determine the antibody and neutralization levels required for protection against infections.

## Figures and Tables

**Table 1:** Baseline Characteristics of all study participants. Frequencies and percentages are given for nominal and ordinal variables. For numerical variables median and range were calculated.

Characteristics	All LTRs at Baseline *n* = 34 *n* (%)/Median (Range)
Age (years)	56 (28–86)
Females	12 (35.3%)
BMI (kg/m^2^)	23.6 (17–37)
Time since transplantation (years)	9.5 (0–28)
Risk factors for severe COVID-19	
Diabetes	6 (17.6%)
Arterial hypertension	20 (58.8%)
Age > 60 years	15 (44.1%)
eGFR < 45 mL/min	14 (41.1%)
BMI > 30 kg/m^2^	6 (17.6%)
2 risk factors	10 (29.4%)
≥3 risk factors	8 (23.5%)
Charlson comorbidity index	5 (1–9)
Vaccination status at baseline	
2 doses	2 (5.9%)
3 doses	5 (14.7%)
4 doses	17 (50%)
5 doses	10 (29.4%)
Median number of prior vaccine doses	4 (2–5)
Prior Infections	
Previously infected	31 (91.2%)
>1 prior infection	3 (8.8%)
Immunosuppression	
Monotherapy	1 (2.9%)
2 Immunosuppressants	18 (52.9%)
≥3 Immunosuppressants	15 (44.1%)

Abbreviations: LTRs: liver transplant recipients; BMI: body mass index; eGFR: estimated glomerular filtration rate.

## Data Availability

Individual participant data will not be shared.

## Supplementary Materials

The following supporting information can be downloaded at https://www.mdpi.com/article/10.3390/v16121942/s1, Supplementary Figure S1: (A) Correlation of Wuhan anti-S and anti-Omicron IgG, (B) Correlation of XBB.1.5 and JN.1 NT50; Supplementary Figure S2: (A) Wuhan-Hu-1 S-specific IgG, (B) Anti-S Omicron IgG; Supplementary Figure S3: Neutralisation in all LTR (n = 34); Supplementary Table S1: Comparison of LTRs with only vaccination and LTRs with COVID-19 following vaccination; Supplementary Table S2: Comparison of LTRs with and without prior SARS-CoV-2 infection; Supplementary Table S3: Comparison of LTRs with and without an immune response to the XBB.1.5pp in the neutralization assay following vaccination.
