# Supplementary material for: Immune Response to SARS-CoV-2 XBB.1.5 and JN.1 Variants Following XBB.1.5 Booster Vaccination in Liver Transplant Recipients
Source: Viruses. 2024 Dec 19;16(12):1942. doi: 10.3390/v16121942 (PMC11680162; doi:10.3390/v16121942)
Supplement: Supplementary file 1 [file viruses-16-01942-s001.zip › viruses-3295777-supplementary.pdf]

Supplementary Table S1: Comparison of LTRs with only vaccination and LTRs with COVID-19 following vaccination

**Characteristics**	**LTRs with vaccination *n*=28 median (range) / n (%)**	**LTRs with COVID-19 following vaccination *n*=6 median (range) / n (%)**	***p*-value**
**Age (years)**	59 (28-86)	44.5 (32-69)	0.098
**Females**	9 (32.1%)	3 (50%)	0.641
**BMI (kg/m²)**	23.6 (17-37)	22.8 (20-34)	0.487
**Time since transplantation (years)**	11 (0-28)	0.5 (0-27)	0.165
**Risk factors for severe COVID-19**			
Diabetes	5 (17.9%)	1 (16.7%)	1.000
Arterial hypertension	18 (64.3%)	2 (33.3%)	0.202
Age >60 years	14 (50%)	1 (16.7%)	0.196
eGFR <45 ml/min	13 (46.4%)	1 (16.7%)	0.364
BMI >30 kg/m²	4 (14.3%)	2 (33.3%)	0.281
Charlson comorbidity index	5 (1-9)	3 (3-4)	**0.011**
**Vaccination status at baseline**			
2 doses	2 (7.1%)	0 (0%)	1.000
3 doses	2 (7.1%)	3 (50%)	**0.028**
4 doses	15 (53.6%)	2(33.3%)	0.656
5 doses	9 (32.1%)	1 (16.7%)	0.644
Median number of prior vaccine doses	4 (2-5)	3.5 (3-5)	0.205
**Prior Infections**			
Previously infected	23 (82.1%)	5 (83.3%)	1.000
>1 prior infection	1 (3.6%)	1 (16.7%)	0.326
**Immunosuppression**			
Monotherapy	1 (3.6%)	0 (0%)	1.000
2 Immunosuppressants	16 (57.1%)	2 (33.3%)	0.387
≥3 Immunosuppressants	11 (39.3%)	4 (66.7%)	0.370
**Serology**			
Wuhan anti-S IgG pre vaccination (BAU/ml)	945 (15.4-8424)	613.3 (125-1214)	0.273
Wuhan anti-S IgG post vaccination (BAU/ml)	3595 (35.7-11520)	3735 (593.1-11520)	0.853
Anti-Omicron IgG pre vaccination (RU/ml)	110 (0.1-1200)	73.8 (8.4-176.9)	0.281
Anti-Omicron IgG post vaccination (RU/ml)	555.6 (9.4-1200)	589 (75.1-1200)	0.842

**NT50 pre-vaccination**			
B.1	121.7 (1-1416)	77.6 (1-206.2)	0.340
XBB.1.5	16.7 (1-361.7)	1 (1-6.3)	**0.026**
JN.1	14.9 (1-352.3)	1 (1-1)	**0.036**
**NT50 post-vaccination**			
B.1	448.6 (1-8015)	402.2 (15-4336)	0.639
XBB.1.5	219.3 (1-3695)	98.1 (1-1208)	0.291
JN.1	142.9 (1-1544)	97.6 (1-748.1)	0.485
**Fold change NT50**			
B.1	2.7 (0.8-55.6)	3.2 (1.7-100.6)	0.501
XBB.1.5	9.1 (1-3036)	60.5 (1-1208)	0.603
JN.1	8.9 (1-872.8)	97.6 (1-748.1)	0.458

Supplementary Table S1: Comparison of LTRs with vaccination and LTRs with COVID-19 following vaccination. Frequencies and percentages are given for nominal and ordinal variables and median and range for numerical variables. Statistical analysis was performed with Fisher's exact test or Mann–Whitney U test. A *p*-value ≤0.05 was considered significant and highlighted in bold. Abbreviations.: LTRs: liver transplant recipients, BMI: body mass index, eGFR: estimated glomerular filtration rate, anti-S: anti-spike, IgG: immunoglobulin G, BAU: binding antibody units, RU: relative units, NT50: neutralization titer 50.

Supplementary Table S2: Comparison of LTRs with and without prior SARS-CoV-2 infection

Characteristics	LTRs without previous infection *n*=3 median (range) / n (%)	LTRs with previous infection *n*=31 median (range) / n (%)	*p*-value
**Age (years)**	60 (45-82)	56 (28-86)	0.552
**Females**	1 (33.3%)	11 (35.5%)	1.000
**BMI (kg/m²)**	18 (17-31.9)	23.8 (17.5-37)	0.294
**Time since transplantation (years)**	20 (11-28)	8 (0-27)	0.067
**Risk factors for severe COVID-19**			
Diabetes	1 (33.3%)	5 (16.1%)	0.453
Arterial hypertension	2 (66.7%)	18 (58.1%)	1.000
Age >60 years	1 (33.3%)	14 (45.2%)	1.000
eGFR <45 ml/min	2 (66.7%)	12 (38.7%)	0.555
BMI >30 kg/m²	0 (0%)	6 (19.4%)	1.000
Charlson comorbidity index	7 (5-9)	4 (1-8)	0.067
**Vaccination status at baseline**			
2 doses	1 (33.3%)	1 (3.2%)	1.000
3 doses	0 (0%)	5 (16.1%)	1.000
4 doses	1 (33.3%)	16 (51.6%)	1.000
5 doses	1 (33.3%)	9 (29.0%)	1.000
Median number of prior vaccine doses	4 (4-5)	4 (2-5)	0.067
**Immuno-suppression**			
**Monotherapy**	0 (0%)	1 (3.2%)	1.000
2 Immunosuppressants	2 (66.7%)	16 (51.6%)	1.000
≥3 Immunosuppressants	1 (33.3%)	14 (45.2%)	1.000
**Serology**			
Wuhan anti-S IgG pre vaccination (BAU/ml)	131.4 (96-199.4)	977.8 (15.4-1200)	**0.014**
Wuhan anti-S IgG post vaccination (BAU/ml)	226.4 (35.7-1359)	3934 (410.4-11520)	**0.007**
Anti-Omicron IgG pre vaccination (RU/ml)	5 (0.1-16.6)	110.6 (50.5-1200)	**0.005**
Anti-Omicron IgG post vaccination (RU/ml)	25.7 (9.4-249.1)	591.2 (368.6-1200)	**0.009**
**NT50 pre-vaccination**			
B.1	6.8 (1-63.0)	131.5 (1-1416)	0.056
XBB.1.5	1 (1-1)	12.8 (1-361.7)	0.144
JN.1	1 (1-1)	1 (1-41.5)	0.237

**NT50 post-vaccination**			
B.1	49.6 (1-68.1)	538.4 (1-8015)	**0.018**
XBB.1.5	1 (1-1)	235.5 (1-3695)	**0.005**
JN.1	1 (1-1)	162.1 (1-1544)	**0.005**
**Fold change NT50**			
B.1	1 (0.8-10.0)	2.7 (1-100.6)	0.195
XBB.1.5	1 (1-1)	13.98 (1-3036)	**0.005**
JN.1	1 (1-1)	19.12 (1-872.8)	**0.005**

Supplementary Table S2: Comparison of LTRs with and without prior SARS-CoV-2 infection. Frequencies and percentages are given for nominal and ordinal variables and median and range for numerical variables. Statistical analysis was performed with Fisher's exact test or Mann–Whitney U test. A *p*-value ≤0.05 was considered significant and highlighted in bold. Abbreviations.: LTRs: liver transplant recipients, BMI: body mass index, eGFR: estimated glomerular filtration rate, anti-S: anti-spike, IgG: immunoglobulin G, BAU: binding antibody units, RU: relative units, NT50: neutralization titer 50.

Supplementary Table S3: Comparison of LTRs with and without an immune response to the XBB.1.5pp in the neutralization assay following vaccination

Characteristics	LTRs without response to XBB.1.5pp *n*=6 median (range) / n (%)	LTRs with response to XBB.1.5pp *n*=28 median (range) / n (%)	*p*-value
**Age (years)**	64.5 (32-82)	55 (28-86)	0.358
**Females**	2 (33.3%)	10 (35.7%)	1.000
**BMI (kg/m²)**	20.6 (17-32.3)	24.0 (17.5-37)	0.370
**Time since transplantation (years)**	21 (0-28)	8 (0-24)	**0.039**
**Risk factors for severe COVID-19**			
**Diabetes**	2 (33.3%)	4 (14.3%)	0.281
**Arterial Hypertension**	5 (83.3%)	15 (53.6%)	0.364
**Age >60 years**	4 (66.7%)	11 (39.3%)	0.370
**eGFR <45 ml/min**	3 (50.0%)	11 (39.3%)	0.672
**BMI >30 kg/m²**	2 (33.3%)	4 (14.3%)	0.281
**Charlson comorbidity index**	6 (3-9)	4.5 (1-8)	0.200
**Vaccination status at baseline**			
**2 doses**	0 (0.0%)	2 (7.1%)	1.000
**3 doses**	0 (0.0%)	5 (17.9%)	0.559
**4 doses**	3 (50.0%)	14 (50.0%)	1.000
**5 doses**	3 (50.0%)	7 (25.0%)	0.328
**Median number of prior vaccine doses**	4.5 (4-5)	4 (2-5)	0.171
**Prior Infections**			
**Previously infected**	3 (50.0%)	28 (100%)	**0.003**
**>1 prior infection**	0 (0.0%)	3 (10.7%)	1.000
**Immunosuppression**			
**Monotherapy**	0 (0.0%)	1 (3.6%)	1.000
**2 Immunosuppressants**	3 (50.0%)	15 (53.6%)	1.000
**≥3 Immunosuppressants**	3 (50.0%)	12 (42.9%)	1.000
**Serology**			
**Wuhan anti-S IgG pre vaccination (BAU/ml)**	128.2 (35.6-235.3)	1161 (15.4-8424)	**0.001**
**Wuhan anti-S IgG post vaccination (BAU/ml)**	501.7 (35.7-1359)	4075 (460.7-11520)	**0.001**
**Anti-Omicron IgG pre vaccination (RU/ml)**	6.7 (0.1-38.6)	144.0 (12.0-1200)	**0.001**
**Anti-Omicron IgG post vaccination (RU/ml)**	117.4 (9.4-249.1)	775.3 (110.9-1200)	**0.001**
**Neutralization Assay B.1**			

**NT50 pre vaccination**	3.9 (1-63.0)	154.3 (1-1416)	**0.001**
**NT50 post vaccination**	32.3 (1-68.1)	576.0 (41.9-8015)	**0.001**
**Fold change NT50**	1.3 (0.8-15.0)	3.0 (1.1-100.6)	0.130

Supplementary Table S3: Comparison of LTRs with and without an immune response to the XBB.1.5pp in the neutralization assay following vaccination. Frequencies and percentages are given for nominal and ordinal variables and median and range for numerical variables. Statistical analysis was performed with Fisher's exact test or Mann–Whitney U test. A *p*-value ≤0.05 was considered significant and highlighted in bold. Abbreviations: LTRs: liver transplant recipients, BMI: body mass index, eGFR: estimated glomerular filtration rate, anti-S: anti-spike, IgG: immunoglobulin G, BAU: binding antibody units, RU: relative units, NT50: neutralization titer 50.

Supplementary Figure S1

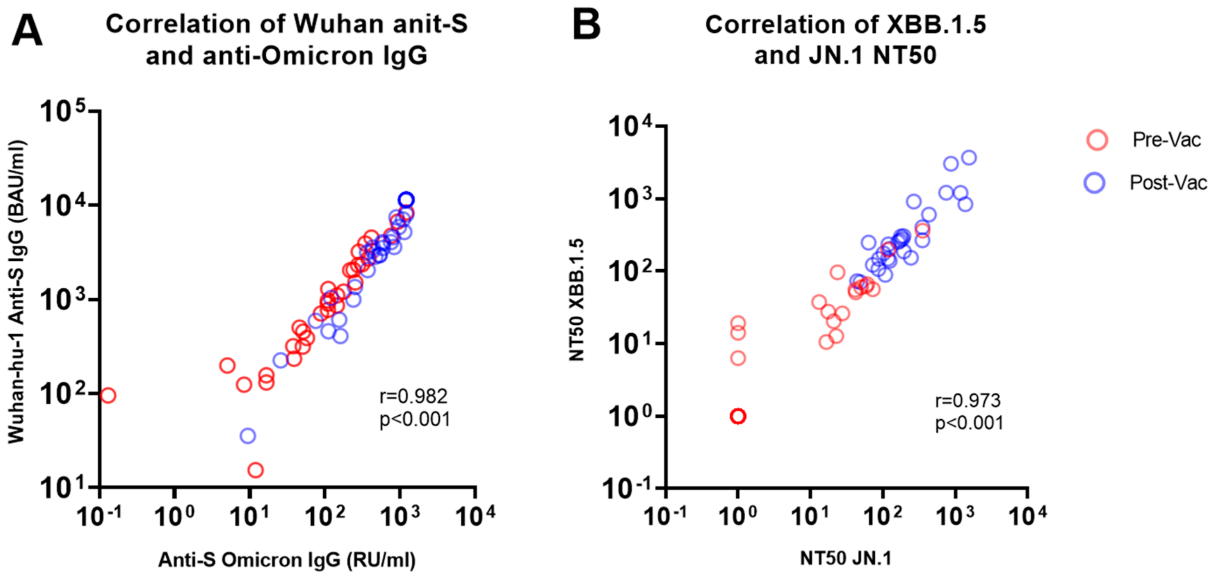

Supplementary Figure S1: (A) Correlation of Wuhan anti-S and anti-Omicron IgG before (red circles) and after (blue circles) XBB.1.5 vaccination (B) Correlation of XBB.1.5 and JN.1 pseudovirus particles neutralization titer 50 before (red circles) and after (blue circles) XBB.1.5 vaccination. Statistical significance was assessed by Spearman rank correlation. Abbreviations: IgG: immunoglobulin G, BAU: binding antibody units, RU: relative units, NT50: neutralization titer 50; S: Spike.

Supplementary Figure S2

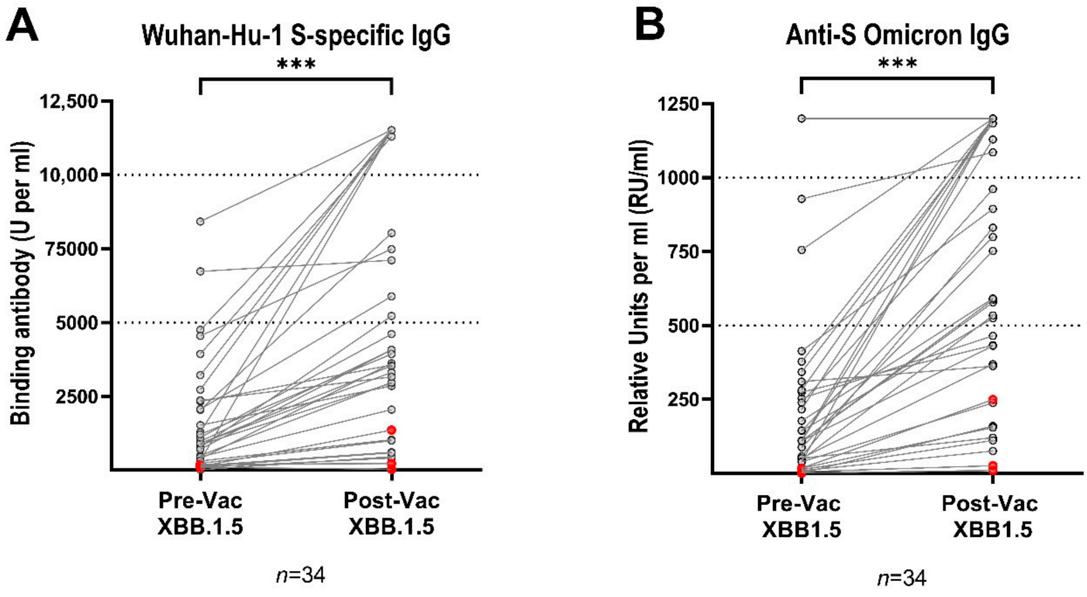

Supplementary Figure S2: (A-B) Concentrations of Wuhan-Hu-1 S-specific IgG and Omicron S-specific IgG in plasma taken before or after vaccination with the BNT162b2 Omicron XBB.1.5 vaccine or after vaccination and infection. Black circles represent individual LTRs. Patients with no prior infection were marked red (n=3). Statistical significance was assessed by paired t-test. (*** = p<0.001). Abbreviations: IgG: immunoglobulin G; Vac: vaccination; S: spike.; U: Units.

Supplementary Figure S3

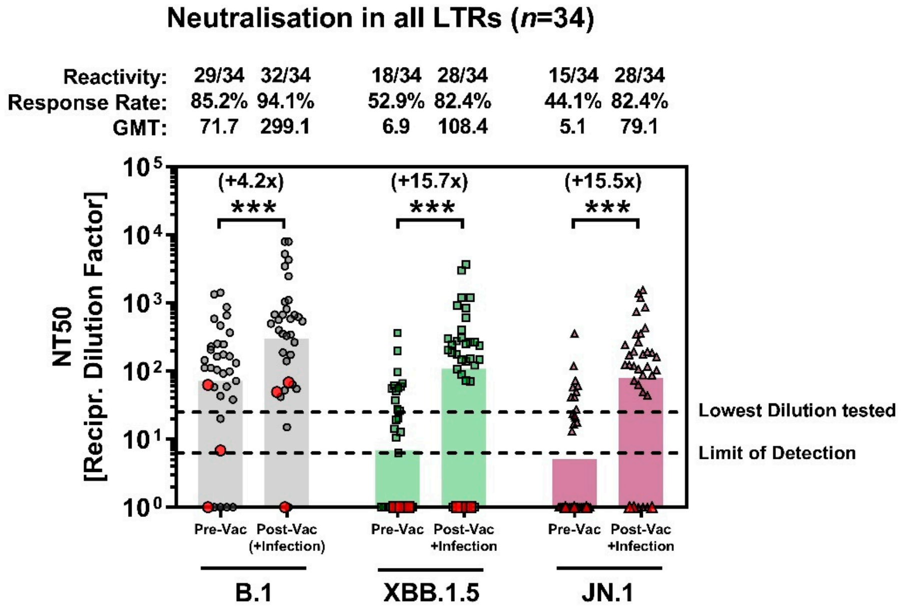

Supplementary Figure S3: Analysis of the neutralization capacity of antibodies before and after XBB.1.5 vaccination against B.1, XBB.1.5, and JN.1 in LTRs. Patients with no prior infection were marked red (n=3). The lowest plasma dilution tested (dashed lines) and the threshold (lower limit of detection) are indicated. Information on response rates and median fold change in neutralization after vaccination are indicated above the graphs. Statistical significance was assessed by Wilcoxon Signed -Rank Test. Of note, for graphical reasons, plasma samples yielding an NT50 value below 6·25 (limit of detection) were manually set at bottom of the axis. Grey dots, green squares, and purple triangles represent the individual responses of LTRs to B.1, XBB.1.5, and JN.1, respectively. Columns indicate the geometric mean NT50 values. Abbreviations: LTRs: liver transplant recipients; GMT: geometric mean titer; Recipr.: reciprocal; NT50: neutralization titer 50.
